# A High Frequency Geometric Focusing Transducer Based on 1-3 Piezocomposite for Intravascular Ultrasound Imaging

**DOI:** 10.1155/2017/9327270

**Published:** 2017-09-05

**Authors:** Xiaohua Jian, Zhile Han, Pengbo Liu, Jie Xu, Zhangjian Li, Peiyang Li, Weiwei Shao, Yaoyao Cui

**Affiliations:** Suzhou Institute of Biomedical Engineering and Technology, Chinese Academy of Sciences, Suzhou, China

## Abstract

Due to the small aperture of blood vessel, a considerable disadvantage to current intravascular ultrasound (IVUS) imaging transducers is that their lateral imaging resolution is much lower than their axial resolution. To solve this problem, a single-element, 50 MHz, 0.6 mm diameter IVUS transducer with a geometric focus at 3 mm was proposed in this paper. The focusing transducer was based on a geometric-shaped 1-3 piezocomposite. The impedance/phase, pulse echo, acoustic intensity field, and imaging resolution of the focusing transducer were tested. For comparison, a flat IVUS transducer with the same diameter and 1-3 piezocomposite was made and tested too. Compared with their results, the fabricated focusing transducer exhibits broad bandwidth (107.21%), high sensitivity (404 mV), high axial imaging resolution (80 *μ*m), and lateral imaging resolution (100 *μ*m). The experimental results demonstrated that the high frequency geometric focusing piezocomposite transducer is capable of visualizing high axial and lateral resolution structure and improving the imaging quality of related interventional ultrasound imaging.

## 1. Introduction

Intravascular ultrasound (IVUS) allows us to see a coronary artery from the inside-out, which has evolved to an important research tool of modern invasive cardiology [1]. In order to get high resolution image, an IVUS transducer usually has a high center frequency (20~60 MHz) [2], like 20 MHz IVUS (Eagle Eye, Volcano Corporation), 40 MHz IVUS (OptiCross, Boston Scientific), and 60 MHz IVUS (Kodama, ACIST Medical Systems). A limitation to current IVUS transducer is that their lateral imaging resolution (200~300 *μ*m) is much lower than their axial resolution (40~100 *μ*m) [3, 4]. This is mainly caused by its small dimension [5, 6], which is extremely limited by the blood vessel.

Usually higher frequency transducer can provide higher lateral resolution, but it will cause a higher attenuation and decrease penetration capability. For example, to get 100 *μ*m lateral resolution at 3 mm, the central frequency of a 0.6 mm diameter IVUS transducer needs to be more than 100 MHz, so it is not a very efficient method. Another possible method to a single-element IVUS transducer is synthetic aperture focusing, which has been shown to be able to improve the imaging resolution and SNR outside focus area by focusing the received signal from several emissions for rotating movements [7]. But it requires large sum data processing and decreases the imaging frame rate. Therefore, it is beneficial to try focusing transducer to get high lateral resolution IVUS image. The traditional method is to use acoustic lens, but fabricating a suitable acoustic lens to focus the ultrasound on IVUS is particularly challenging, since the IVUS catheter outer diameter is limited in the range of 3F~9F (1 mm~3 mm), and the focusing length should be smaller than the coronary artery diameter (3~5 mm).

Therefore, self-focusing transducer will be a good choice. Fresnel Half-Wave-Band sources method was widely used for self-focusing, but its radius will be larger than 3 mm for getting better performance with enough loops at 20~60 MHz [8]. One possible method is to fabricate high frequency PMN-PT single crystal focusing transducer by a mechanical dimpling technique. The reported dimpled 30 MHz single crystal focusing transducer with a diameter of 1.6 mm can prove 139 *μ*m lateral resolution [9]. Similarly, oblong-shaped focused IVUS transducers using PZT were also able to improve the lateral resolution [10]. Angled-focused single-element transducer was another choice to improve IVUS lateral imaging resolution. As reported, the lateral resolution was improved from 270 *μ*m to 120 *μ*m with the angled-focused 45 MHz PMN-PT single-element transducer [11]. But the single crystal or PZT are fragile and easy to crack in the process, which would affect the transducer performance and yield, while 1-3 piezocomposite will be an alternative choice for the active material, which can have high frequency, low acoustic impedance, and wide bandwidth [12, 13]. It has been reported for IVUS imaging and other high frequency endoscopic ultrasound imaging research [14–16]. There are many advantages to use a 1-3 piezocomposite as the IVUS transducer active substrate. First of all, the acoustic impedance of a 1-3 piezocomposite is significantly lower than common pure bulk ceramic, which can effectively decrease the acoustic mismatch between transducer and tissue and avoid the need for more matching layers [17, 18]. Secondly, the electromechanical coupling coefficient of 1-3 composite is much higher than common piezoelectric ceramic, which is helpful to improve the imaging sensitivity. Furthermore, 1-3 piezocomposite consists of a large percentage epoxy, which made it easy to be geometrically shaped. Therefore, 1-3 piezocomposite was suitable for making geometric focusing IVUS transducer. Even in an early patent, the related proposal has been described [14]. Therefore, in this paper we proposed a geometric focusing 50 MHz piezocomposite transducer for intravascular ultrasound imaging. For comparing, a flat IVUS transducer with the same 1-3 piezocomposite was also fabricated and tested following the same experiments; the results are described below in more detail.

## 2. Design and Fabrication

The focal length of the transducer was designed to 3 mm, which is close to the natural focus *F* corresponding to the near field range of flat IVUS transducer [5]:

(1) $$\begin{array}{c} F=\frac{D^{2}}{4\lambda }, \end{array}$$

where the diameter *D* of the transducer is 0.6 mm, the wavelength *λ* is about 30 *μ*m in our situation, and then the natural focal length of flat IVUS transducer is about 3 mm.

The first step in the fabrication procedure was to make a high frequency 1-3 piezocomposite. Considering our laboratory experimental facilities, the design parameters of the 1-3 composite were listed in Table 1.

A two-dice and filling process was applied to fabricate this 1-3 composite. Firstly, a grid pattern was diced into a ceramic PZT-5H with 500 *μ*m thickness using a 12 *μ*m dicing saw. The depth of cuts is about 120 *μ*m, and the pitch is 60 *μ*m. The cuts were then filled with epoxy (Epo-Tek 301-2, Epoxy Technologies, Billerica, MA). After curing, the second set of cuts was made through the center of the PZT-5H pillars to create a composite pattern with 18 *μ*m pillars and 12 *μ*m kerfs as designed. The composite was filled and cured with Epo-Tek 301-2 again and then lapped to 33 *μ*m thick. After that, a 200 Å chrome/gold layer as the electrode was sputtered.

**Figure 1:**
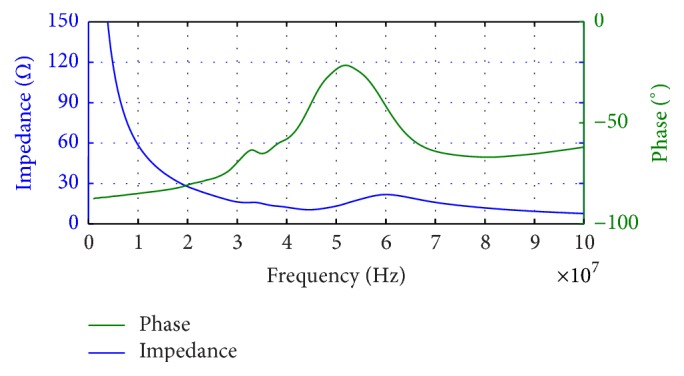
The measured impedance/phase of fabricated 1-3 piezocomposite.

The electrical impedance of fabricated 1-3 piezocomposite was measured with an impedance analyzer E4991A (1 MHz~3 GHz, Agilent Technologies, USA), just as Figure 1 shows. The measured center frequency is 52.76 MHz.

Its electromechanical coupling coefficient *K*_*t*_ can be calculated as [15]

(2) $$\begin{array}{c} K_{t}=\sqrt{\frac{\pi }{2}\times \frac{f_{s}}{f_{p}}\times \tan\left(\frac{\pi }{2}\times \frac{f_{p}-f_{s}}{f_{p}}\right)}, \end{array}$$

where *f*_*p*_ is the parallel resonant frequency at which the resistance reaches the maximum and *f*_*s*_ is the resonant frequency at which the conductance reaches the minimum. For our sample, *f*_*s*_ is 44.05 MHz and *f*_*p*_ is 60.36 MHz, so according to (2), *K*_*t*_ is about 0.70, which was higher than pure bulk ceramic (~0.5).

**Figure 2:**
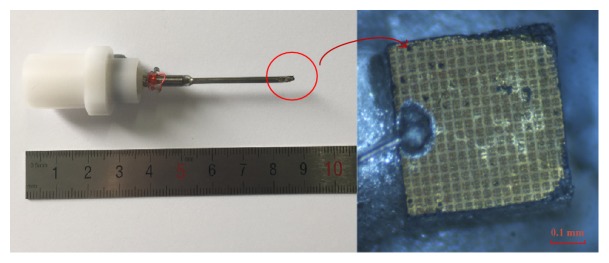
The fabricated focusing IVUS transducer.

In the shaping process, the composite was firstly heated at 60°C for 1 hour to make it more flexible and then quickly mounted on a 3 mm radius PTFE ball with wax. The PTFE ball was used as the curving jig, which decides the focal length of the transducer. Conductive silver epoxy E-Solder 3022 was applied to the composite as the backing material in a PDMS (polydimethylsiloxane) mold. After curing at room temperature, the sample was heated to remove the PTFE ball. Then the sample was diced into the size of 0.6 mm × 0.6 mm. The individual piece was placed in a 1.2 mm diameter needle housing; the center core and mesh wire of a coaxial wire were connected to the piezocomposite surface and backing layer, respectively, with silver conductive. The gap between the transducer and the stainless steel needle was filled in by an insulating epoxy. At last, a 9 *μ*m Parylene C layer was coated as its matching layer. The final fabricated focusing transducer was shown in Figure 2.

## 3. Results and Discussion

**Figure 3:**
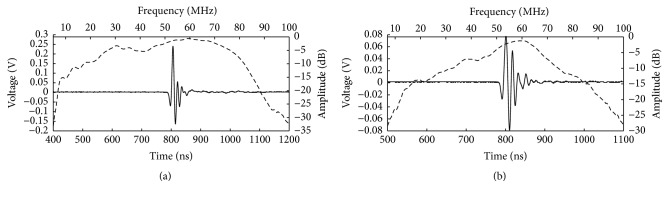
Pulse echo response and spectrum of (a) fabricated focusing IVUS transducer and (b) fabricated flat IVUS transducer.

A DPR500 (pulse amplitude: 90 V, gain: 0 dB, filter: 5~300 MHz, RPF: 200 Hz, JSR Ultrasonics, USA) was used as the pulser-receiver to measure the center frequency, −6 dB bandwidth, and pulse echo amplitude of the fabricated focusing transducer and flat transducer. The pulse echo response was measured by recording the reflection from a quart polyethylene plastics flat placed at 3 mm in front of the transducer. The measured center frequency is 51.78 MHz, the −6 dB bandwidth is 107.21%, and the pulse half width is 17.37 ns just as Figure 3(a) shows. And the transducer pulse echo amplitude was measured as 404 mV with 50 Ω coupling impedance setting. For comparison, a flat IVUS transducer (unfocused) with the same diameter 0.6 mm was fabricated. This flat transducer was composed of the same 1-3 piezocomposite material, a Parylene C matching layer, and E-Solder 3022 backing layer. According to its pulse echo waveform and corresponding frequency spectrum as shown in Figure 3(b), its measured center frequency, −6 dB bandwidth, pulse half width, and amplitude were 53.77 MHz, 64.77%, 29.86 ns, and 156 mV, respectively.

**Figure 4:**
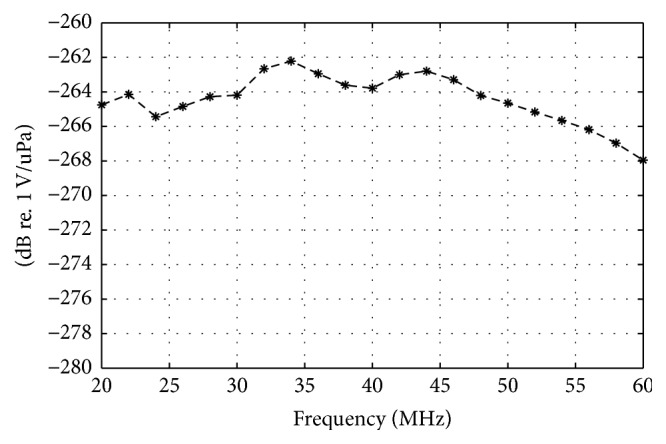
The response of HGL-0085 hydrophone with special calibration in the range of 20~60 MHz. Measurement uncertainty: 20~40 MHz, 2.2 dB; 40~60 MHz, 3.0 dB.

The acoustic distribution was measured by a 3D scanning system UMS III (scan step resolution 0.001 mm, Precision Acoustics Ltd., Dorchester, UK) with a specially calibrated HGL-0085 hydrophone (20~60 MHz), as shown in Figure 4.

**Figure 5:**
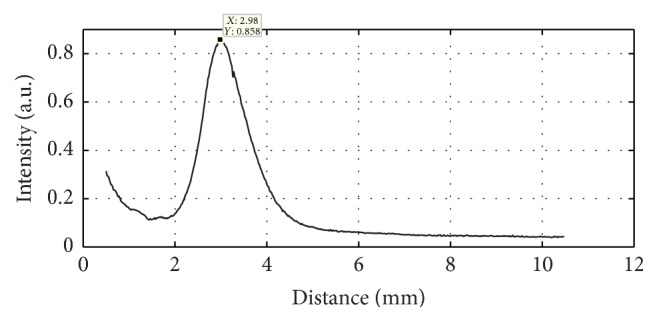
The acoustic intensity distribution of fabricated focusing IVUS transducer along *z*-axis.

The hydrophone measurement step size was 30 *μ*m. The acoustic intensity distribution of this fabricated transducer along *z*-axis was measured as shown in Figure 5. For avoiding the collision between the tested hydrophone and transducer, the recorded data was beginning at 0.5 mm away from the transducer. The measured focal length along the axial direction is 2.98 mm, which is very close to the designed focal length 3 mm. While the intensity of a flat IVUS transducer oscillates sharply in the near field range, then because of the attenuation, it will decrease linearly with the distance.

**Figure 6:**
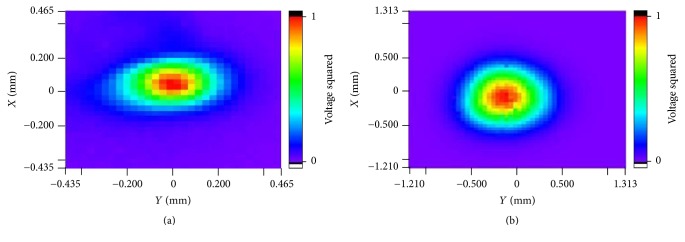
Measured acoustic intensity distribution of (a) fabricated focusing IVUS transducer; (b) fabricated flat IVUS transducer in the natural focus plane (*X*-*Y* plane).

Because the lateral resolution is determined by the beam width perpendicular to the direction of wave propagation in an imaging plane, the transducer's acoustic intensity distributions in the focal plane (*X*-*Y* plane) were scanned with UMS III. According to the focal plane result in Figure 6(a), the diameter of the focal point of the fabricated focusing IVUS transducer (at −6 dB) is about 100 *μ*m, which is one-third of the flat IVUS transducer's 300 *μ*m, just as Figure 6(b) shows. These results indicate that the focusing IVUS transducer can achieve a higher lateral imaging resolution than usual flat ones.

**Figure 7:**
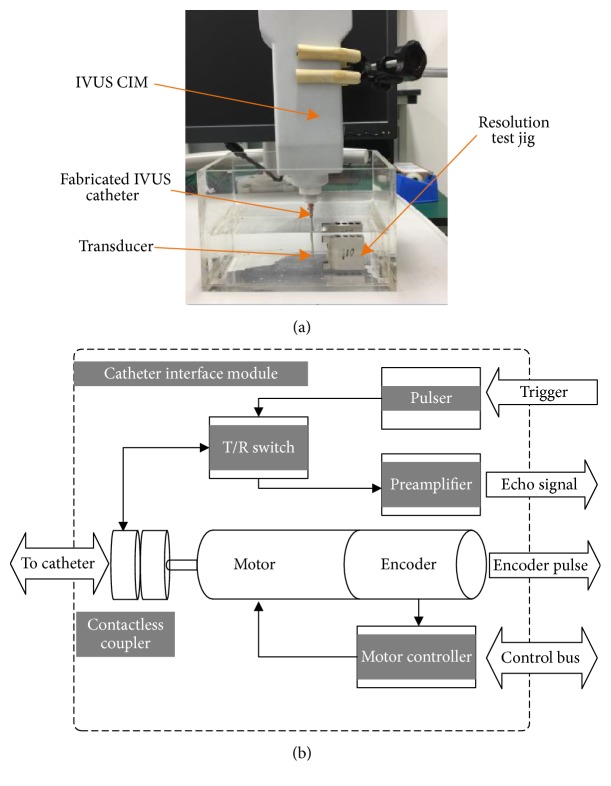
The imaging resolution test system: (a) experimental setup and (b) the block diagram of IVUS CIM (catheter interface module) [16].

The imaging resolution was tested by our IVUS system [16]. The transducer was fixed on the top of a catheter, and the catheter is driven by a motor, which is in the IVUS catheter interface module (CIM), to do a rotary scan. The block diagram of CIM is shown in Figure 7(b). A single-element rotary scanning catheter is utilized to fix and rotate the transducer, which can spin at a speed of 1800 RPM. In the CIM, a contactless coupler with a flatten transfer curve from 10 to 110 MHz with an attenuation better than −1 dB is designed and manufactured to transfer high frequency signals between rotary and stationary side. For each cycle the catheter have turned, a frame composed of 512 scan lines would be captured. 12 bits analog to digital converter rate with a sampling rate of 220 MSPS ensure the echo information in enough frequency range could be recorded and provide a 25 fps imaging speed. More details of our IVUS system can be found in our published [16].

**Figure 8:**
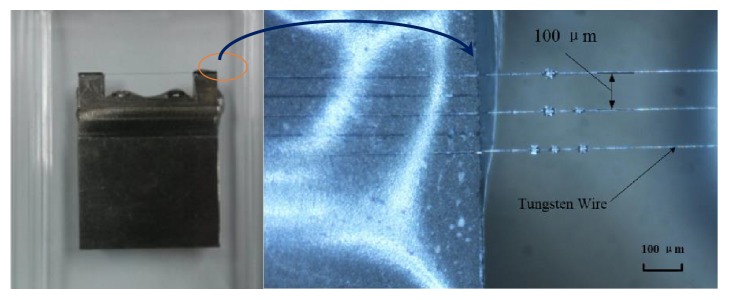
The imaging resolution test jig and tungsten wire phantom (100 *μ*m).

The imaging targets were some resolution test jigs with fixed interval tungsten wires, including 50 *μ*m, 60 *μ*m, 70 *μ*m, 80 *μ*m, 90 *μ*m, 100 *μ*m, 200 *μ*m, 300 *μ*m, and 400 *μ*m, just as Figure 8 shows. The diameter of tungsten wires is 10 *μ*m. When testing the axial resolution, the transducer was adjusted to be perpendicular to the wire phantom, while for lateral resolution testing it will be parallel to the wire phantom, just as Figure 7(a) shows. For getting the best lateral resolution, the distance between the phantom wires and transducer was set to be 3 mm just as the focal length.

**Figure 9:**
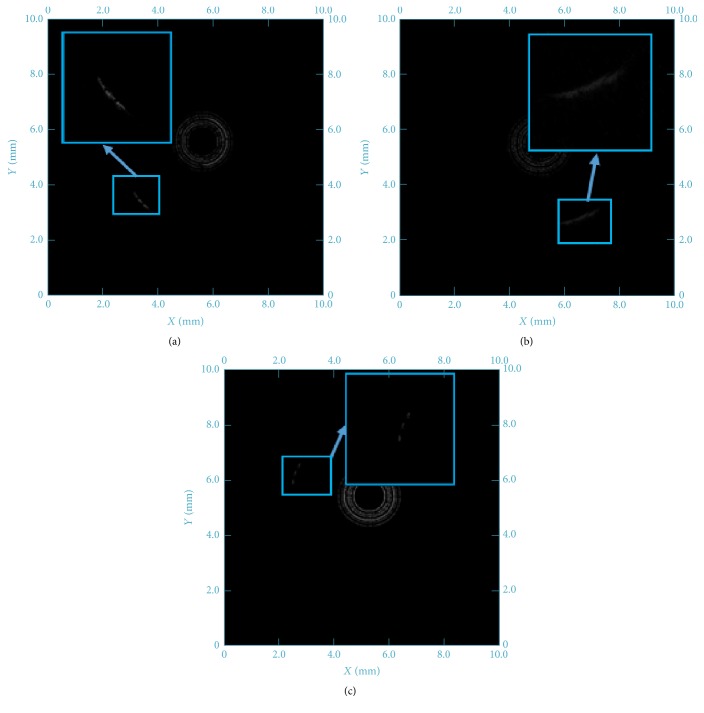
The lateral imaging resolution test images of (a) 100 *μ*m interval tungsten wire phantoms with focusing IVUS transducer; (b) 100 *μ*m interval tungsten wire phantoms with flat IVUS transducer; (c) 300 *μ*m interval tungsten wire phantoms with flat IVUS transducer.

Figure 9 showed the lateral resolution testing results of the fabricated focusing and flat IVUS transducer, respectively. The bright arcs in the images represent the tungsten wires, while the center black hole and rings are caused by the transducer rotating. When using the 100 *μ*m interval tungsten wire phantom, the image of three tungsten wires is only a blurred curve in Figure 9(b) detected by the flat transducer. It is impossible to distinguish each line in this image. Until the 300 *μ*m interval tungsten wire phantom was used, the lines can be clearly separated in detected image in Figure 9(c). Therefore, the lateral resolution of flat IVUS transducer is tested as 300 *μ*m.

In the same way, the lateral resolution of fabricated focusing IVUS transducer is tested as 100 *μ*m, just as Figure 9(a) showed, which is three times of the flat IVUS transducer. That is because the lateral beam width is greatly reduced by adjusting the focal performance of focused transducer [19]. Therefore, it is beneficial to try focused transducer to get high lateral resolution IVUS image.

**Figure 10:**
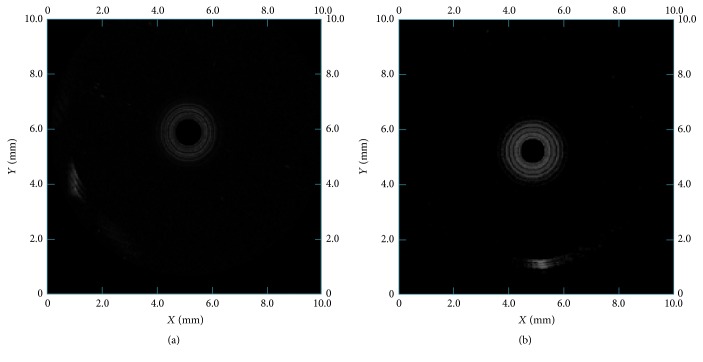
The axial resolution test images of 80 *μ*m interval tungsten wire phantoms with (a) focusing IVUS transducer and (b) flat IVUS transducer.

Similarly, Figures 10(a) and 10(b) showed the axial resolution testing images of the focusing and flat IVUS transducer, respectively. According to the results, the axial resolution of focusing IVUS transducer is 80 *μ*m, and the flat IVUS transducer is 80 *μ*m too. That is because the axial imaging resolution is mainly decided by center frequency of the transducer.

**Figure 11:**
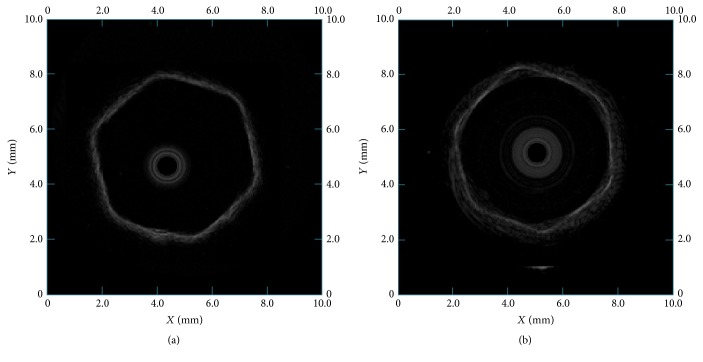
The image of hexagon phantom detected by (a) focusing IVUS transducer and (b) flat IVUS transducer.

Furthermore, a hexagon hole phantom with 3 mm side length was used to test the imaging performance. The phantom was made by mixing 9 *μ*m silicon carbide (2% by weight) and 3 *μ*m aluminum oxide (2% by weight) with PDMS. When testing, the hexagon hole was filled with degas water, and the catheter distal with transducer was immersed into the hole.  Figure 11 shows the phantom images detected by the fabricated focusing and flat IVUS transducer, respectively. Two images were both displayed in 12 dB dynamic range. Because of the high resolution and sensitivity, it is obvious that the image obtained by the focusing IVUS transducer was more clear and in a better shape. Particularly, the average contrast with respect to the locations of boundary and background in the images obtained by focusing and flat IVUS transducer were −13.1 dB and −16.2 dB, respectively.

At last, the main testing results were summarized in Table 2.

## 4. Conclusion

In this study, a micro high frequency IVUS transducer with spherical focusing was successfully produced using PZT/epoxy 1-3 composite. The prototyped focusing IVUS transducer has a small size (0.6 × 0.6 mm), short focal length (3 mm), and high lateral imaging resolution (100 *μ*m). The image obtained by our homemade IVUS system with the fabricated focusing transducer had a high signal to noise ratio and image quality. Based on these results, this micro high frequency focusing transducer has the potential for intravascular ultrasound imaging and various high frequency endoscopic ultrasound imaging.

## Figures and Tables

**Table 1:** The design parameters of 1-3 piezocomposite.

Frequency	Kerf width	Pillar width	Composite thickness	Volume fraction
50 MHz	12 *μ*m	18 *μ*m	33 *μ*m	36%

**Table 2:** The comparison of measured focusing and flat IVUS transducer performance.

Properties	Flat IVUS transducer	Focusing IVUS transducer
Center frequency	53.77 MHz	51.78 MHz
Aperture	0.6 mm	0.6 mm
Bandwidth	64.77%	107.21%
Echo peak	156 mV	404 mV
Axial resolution	80 *μ*m	80 *μ*m
Lateral resolution	300 *μ*m	100 *μ*m

## References

[1] Potkin B. N., Bartorelli A. L., Gessert J. M. (1990). Coronary artery imaging with intravascular high-frequency ultrasound. *Circulation*.

[2] Schoenhagen P., DeFranco A., Nissen S., Tuzcu E. (2005). *IVUS Made Easy*.

[3] Prati F., Regar E., Mintz G. S. (2010). Expert review document on methodology, terminology, and clinical applications of optical coherence tomography: physical principles, methodology of image acquisition, and clinical application for assessment of coronary arteries and atherosclerosis. *European Heart Journal*.

[4] Foster F. S., Ryan L. K., Lockwood G. R. (1993). High Frequency Ultrasound Scanning of the Arterial Wall. *Intravascular Ultrasound*.

[5] Suri J. S., Wilson D. L., Laxminarayan S. (2005). *Handbook of Biomedical Image Analysis*.

[6] Rhee S. High frequency (IVUS) ultrasound transducer technology - Applications and challenges.

[7] Andresen H., Nikolov S. I., Jensen J. A. (2011). Synthetic aperture focusing for a single-element transducer undergoing helical motion. *IEEE Transactions on Ultrasonics, Ferroelectrics, and Frequency Control*.

[8] Huang D., Kim E. S. (2001). Micromachined acoustic-wave liquid ejector. *Journal of Microelectromechanical Systems*.

[9] Chen Y., Lam K. H., Zhou D. (2013). High frequency PMN-PT single crystal focusing transducer fabricated by a mechanical dimpling technique. *Ultrasonics*.

[10] Lee J., Jang J., Chang J. H. (2017). Oblong-Shaped-Focused Transducers for Intravascular Ultrasound Imaging. *IEEE Transactions on Biomedical Engineering*.

[11] Yoon S., Williams J., Kang B. J. (2015). Angled-focused 45 MHz PMN-PT single element transducer for intravascular ultrasound imaging. *Sensors and Actuators, A: Physical*.

[12] Li X., Ma T., Tian J., Han P., Zhou Q., Shung K. K. (2014). Micromachined PIN-PMN-PT crystal composite transducer for high-frequency intravascular ultrasound (IVUS) imaging. *IEEE Transactions on Ultrasonics, Ferroelectrics, and Frequency Control*.

[13] Yuan J. R., Jiang X., Cao P.-J. High frequency piezo composites microfabricated ultrasound transducers for intravascular imaging.

[14] Yuan J., Cao P., Romley R. Piezocomposite transducers.

[15] Safari A., Akdoğan E. K. (2008). Piezoelectric and acoustic materials for transducer applications. *Piezoelectric and Acoustic Materials for Transducer Applications*.

[16] Xiang Y., Xu J., Lv T., Gu T., Han Z., Cui Y. A graphic processing unit based intravascular ultrasound (IVUS).

[17] Brown J. A., Foster F. S., Needles A., Cherin E., Lockwood G. R. (2007). Fabrication and performance of a 40-MHz linear array based on a 1-3 composite with geometric elevation focusing. *IEEE Transactions on Ultrasonics, Ferroelectrics, and Frequency Control*.

[18] Jiang X., Yuan J. R., Cheng A. Microfabrication of piezoelectric composite ultrasound transducers (PC-MUT).

[19] Saijo Y., Van der Steen A. F. W. (2003). *Vascular ultrasound*.

